# Hyperspectral Imaging of Head and Neck Squamous Cell Carcinoma for Cancer Margin Detection in Surgical Specimens from 102 Patients Using Deep Learning

**DOI:** 10.3390/cancers11091367

**Published:** 2019-09-14

**Authors:** Martin Halicek, James D. Dormer, James V. Little, Amy Y. Chen, Larry Myers, Baran D. Sumer, Baowei Fei

**Affiliations:** 1 Department of Bioengineering, The University of Texas at Dallas, Richardson, TX 75080, USA; 2 Department of Biomedical Engineering, Emory University and Georgia Institute of Technology, Atlanta, GA 30329, USA; 3 Department of Pathology and Laboratory Medicine, Emory University School of Medicine, Atlanta, GA 30322, USA; 4 Department of Otolaryngology, Emory University School of Medicine, Atlanta, GA 30322, USA; 5 Department of Otolaryngology, The University of Texas Southwestern Medical Center, Dallas, TX 75390, USA; 6 Advanced Imaging Research Center, The University of Texas Southwestern Medical Center, Dallas, TX 75390, USA; 7 Department of Radiology, The University of Texas Southwestern Medical Center, Dallas, TX 75390, USA

**Keywords:** hyperspectral imaging, head and neck cancer, squamous cell carcinoma, deep learning, convolutional neural network

## Abstract

Surgical resection of head and neck (H and N) squamous cell carcinoma (SCC) may yield inadequate surgical cancer margins in 10 to 20% of cases. This study investigates the performance of label-free, reflectance-based hyperspectral imaging (HSI) and autofluorescence imaging for SCC detection at the cancer margin in excised tissue specimens from 102 patients and uses fluorescent dyes for comparison. Fresh surgical specimens (*n* = 293) were collected during H and N SCC resections (*n* = 102). The tissue specimens were imaged with reflectance-based HSI and autofluorescence imaging and afterwards with two fluorescent dyes for comparison. A histopathological ground truth was made. Deep learning tools were developed to detect SCC with new patient samples (inter-patient) and machine learning for intra-patient tissue samples. Area under the curve (AUC) of the receiver-operator characteristic was used as the main evaluation metric. Additionally, the performance was estimated in mm increments circumferentially from the tumor-normal margin. In intra-patient experiments, HSI classified conventional SCC with an AUC of 0.82 up to 3 mm from the cancer margin, which was more accurate than proflavin dye and autofluorescence (both *p* < 0.05). Intra-patient autofluorescence imaging detected human papilloma virus positive (HPV+) SCC with an AUC of 0.99 at 3 mm and greater accuracy than proflavin dye (*p* < 0.05). The inter-patient results showed that reflectance-based HSI and autofluorescence imaging outperformed proflavin dye and standard red, green, and blue (RGB) images (*p* < 0.05). In new patients, HSI detected conventional SCC in the larynx, oropharynx, and nasal cavity with 0.85–0.95 AUC score, and autofluorescence imaging detected HPV+ SCC in tonsillar tissue with 0.91 AUC score. This study demonstrates that label-free, reflectance-based HSI and autofluorescence imaging methods can accurately detect the cancer margin in ex-vivo specimens within minutes. This non-ionizing optical imaging modality could aid surgeons and reduce inadequate surgical margins during SCC resections.

## 1. Introduction

Surgery is often the primary treatment for head and neck squamous cell carcinoma (HNSCC) [1]. Primary surgery is the modality of choice for resectable oral cavity cancers and late stage disease of the larynx and hypopharynx [2]. Management of locally advanced SCC may also require a multimodal approach with adjuvant chemoradiation therapy [1,3]. Nearly 90% of cancers of the upper aerodigestive track of the head and neck are SCC [4,5]. Depending on the extent of the disease, radiation therapy or chemotherapy alone may be the primary curative modality selected, such can be the case with unresectable, recurrent, or metastatic cancers and also with cases known to be susceptible to chemoradiation [1,2]. Human papilloma virus (HPV) is an identified cause of SCC, and the most common location for HPV positive (HPV+) SCC is the oropharynx, with nearly 60% of oropharyngeal SCC cases being HPV+ [1,6]. Approximately two-thirds of patients with HNSCC present with stage III or IV advanced disease [7]. Adequate surgical removal of the primary SCC is vital to successful patient outcomes, improved quality of life, survival, and reduced recurrence [8,9]. Surgeons can use pre-operative imaging, such as CT or MRI, for planning, but during the surgery, surgeons rely on experience, visual cues, and tactile palpation to determine the extent of the disease. Excised samples and tissue biopsies can be sent for pathological analysis and consultation to determine if the cancer has been sufficiently resected [9,10,11]. 

Intraoperative pathologist consultations (IPCs) can be time-consuming and may not fully reflect the extent of the disease due to limitations in tissue sampling and preparation. While the overall accuracy of frozen-sections in IPCs is upwards of 97%, the accuracy for challenging cases, such as positive and close margins, ranges from 71 to 92%, with sensitivities reported as low as 34 to 77% [10,11,12,13,14]. These errors can compound, leading to reported positive margins in up to 12% and close margins in up to 19% of HNSCC surgeries, despite having negative frozen sections during IPC [10,11,12,13,14]. For example, in oral cavity SCC, up to 30% of patients have positive margins after surgery [3,15].

The task of surgical guidance for SCC resections in the head and neck has been explored with increasing volume in the past five years using several imaging modalities coupled with machine learning [16]. Some methods propose using fluorescently-tagged monoclonal antibodies that require intravenous administration but have specific optical signatures in the near-infrared (NIR) spectrum, with successful outcomes of studies with 21 patients [3,15], and other methods utilize topical fluorescent dyes for targeting SCC [17,18]. Label-free optical imaging methods that utilize only narrow bands in the blue and green visible spectrum have also demonstrated success at delineating oral SCC margins in-vivo in studies with 20 patients [19,20].

Hyperspectral imaging (HSI) is an emerging technology in biomedicine [21] and has been used for cancer detection studies both ex-vivo and in-vivo [22,23]. HSI has been utilized for brain cancer detection in-vivo using machine learning algorithms and an optimized, clinical workflow for neurosurgeons [24,25]. Additionally, HSI has been proposed for laparoscopic cancer detection in colorectal surgeries with demonstrated potential [26,27]. 

Our group reported proof-of-concept studies on HSI for the detection of head and neck SCC in fresh surgical specimens from human patients [17,18]. In our previous pilot studies with HSI, manually selected regions of interest (ROIs) were classified, and image preprocessing was used to remove specular glare pixels before tissue classification [17,18]. In our other works [28,29,30], deep learning algorithms were developed for HSI tissue classification in both whole primary-tumor specimens and at the SCC cancer-normal margin, but only in limited sample sizes from 21 to 29 patients employing cross-validation. Previous works from other groups focus on SCC detection in excised tongue SCC specimens, using both proof-of-concept ROI-based detection of SCC in 7 specimens [31] and tumor semantic segmentation of the entire cancer-margin specimens [32] with promising results in leave-one-patient-out cross-validation experiments of 14 patients.

In this large study of 293 tissue specimens from 102 patients with SCC, we develop deep learning methods to classify the whole tissue specimens instead of ROIs and thus further investigate the full potential of label-free HSI-based imaging methods for SCC detection. This is the first work to conduct fully-independent training, validation, and testing directly of the SCC tumor margin with a large patient dataset (*N* = 102 patients), divided into conventional, keratinizing SCC with variants (*N* = 88) and HPV+ (*N* = 14) SCC cohorts. The tissues represent a variety of anatomical sites to give an accurate assessment of the feasibility of label-free, non-contact, and non-ionizing HSI-based imaging modalities for SCC detection. Additionally, this is the first study to investigate and quantify HSI-based methods for HPV+ SCC detection directly. It is hypothesized that deep learning algorithms can be developed to enable label-free HSI-based methods, namely reflectance-based HSI and autofluorescence imaging, to perform with substantial accuracy to provide meaningful information to guide complete surgical resections. Furthermore, it is hypothesized that label-free HSI-based methods will outperform the fluorescent dye-based methods due to lack of target specificity with sufficient signal-to-noise in SCC tissues. The results of this study will inform if HSI and other fluorescence imaging modalities can be expected to provide specific benefits to cancer margin detection during SCC resection surgeries.

## 2. Materials

### 2.1. Head and Neck SCC Patient Dataset

Patients with head and neck SCC undergoing routine surgery at the Emory University Hospital Midtown (EUHM) were recruited by providing informed, written consent to the research coordinator, who de-identified the patient data. All methods and procedures were approved by the Emory University Institutional Review Board (IRB) under the Head and Neck Satellite Tissue Bank protocol. Fresh, ex-vivo surgical specimens were collected from the surgical pathology laboratory, making sure not to impede routine clinical service. Three tissue samples from each patients’ gross tissue specimen were collected: a tissue specimen of the primary tumor (T), an all normal tissue (N), and a specimen at the tumor-involved cancer margin (TN). The specimens were transported to an imaging laboratory to perform gross-level optical imaging of the ex-vivo specimens for SCC detection [17,18,29]. The median sizes (height × width) of the tissues were 9 × 6 mm, 10 × 7 mm, and 9 × 5 mm for the T, TN, and N tissues, respectively, with an approximate tissue depth of 2 to 3 mm. In total, 293 tissue specimens were collected from 102 patients with head and neck SCC to be included in this study.

A pathologist with expertise in head and neck cancer categorized the tissue samples collected for this study into two groups per cancer subtype: Conventional SCC with variants and HPV+ SCC. The conventional SCC group (*N* = 88 patients) was comprised of conventional, keratinizing SCC (*N* = 85), adenosquamous carcinoma (*N* = 1), basaloid SCC (*N* = 1), and spindle cell SCC (*N* = 1). The HPV+ SCC group consisted of 14 patients that were all identified as p16 marker positive using immunohistochemistry, and one was a neuroendocrine SCC. Table 1 shows the breakdown of tissue samples for different locations of primary tumors along with other patient characteristics and cancer properties. For this study, we defined the oral cavity as broadly consisting of non-tongue structures inside the oral cavity: Oral mucosa (*N* = 3), gingiva (*N* = 5), floor of mouth (*N* = 12), retromolar trigone (*N* = 4), maxillary (*N* = 2), and mandibular surfaces (*N* = 9). Although it is not the clinical standard, tongue specimens were presented separately because it was the single largest anatomical site in this study, and the excised specimens we acquired from the tongue were typically very distinct, both optically and anatomically, with thick surface epithelium and large amounts of skeletal muscle, compared with the rest of the oral cavity cases. The pharynx consisted of cases originating in the oropharynx (*N* = 12 total; 10 HPV+ and 2 HPV-), hypopharynx (*N* = 4), and *N* = 3 cases of HPV+ tonsillar SCC. One patient’s primary location was unknown, only nodal HPV+ neck mass was available.

### 2.2. Hyperspectral Imaging

**Figure 1:**
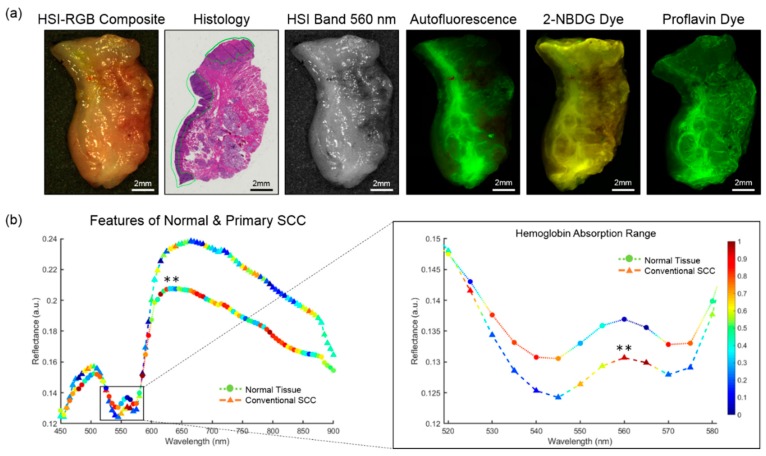
A representative cancer-involved tissue specimen of conventional, keratinizing SCC of the mandibular gingiva. (**a**) From left to right: RGB image made from HSI. The histological image, which serves as the ground truth, has SCC annotated in green. HSI single band at 550 nm. Fluorescence imaging modalities of the same specimen; (**b**) Spectral feature saliency from CNN gradients of correctly classified HSI for conventional SCC and normal upper aerodigestive tract tissues. Left: Full spectra from 450 to 900 nm of SCC and normal tissues. Symbol colors represents the relative, scaled importance of the spectral feature for making the correct prediction of cancer or normal from the HSI (0 is low saliency; 1 is high saliency). Right: Spectral cutout from 520 to 580 nm, corresponding to the hemoglobin range. The double asterisk (**) indicates that statistically significant differences (*p* < 0.01) were observed in reflectance values between SCC and normal for all spectral bands (450 to 900 nm). The most important spectral feature for correctly predicting SCC in HSI was the oxygenated hemoglobin peak at 560 and 565 nm.

The HSI were acquired of the gross-level surgical specimens using a Maestro spectral imaging system (Perkin Elmer Inc., Waltham, Massachusetts), which captured 2D images at full spatial resolution using a 16-bit charge coupled device and stepped through the spectral bands using a liquid crystal tunable filter [17,18,29]. The spatial resolution of the HS sensor was 1040 by 1392 pixels, which corresponds to a specimen-level resolution of 25 µm per pixel. The HSI were captured from 450 to 900 nm in 5 nm spectral bands to produce a hyperspectral data cube (hypercube) with 91 spectral bands. The average imaging time for acquiring a single HSI at this resolution was about one minute. The hyperspectral data were normalized in each spectral band individually by subtracting the inherent dark current (imaging with a closed camera shutter) and dividing by a white reference disk. An RGB composite image was generated from the normalized hypercube by applying a Gaussian kernel in each color region. Figure 1 shows a representative patient tissue specimen and average spectral signatures of SCC and normal tissues.

### 2.3. Fluorescence Imaging

To compare the ability of HSI for cancer margin detection, several optical imaging modalities were acquired afterwards: A second label-free and two dye-based methods [17,18]. Figure 1 shows a representative patient tissue specimen of all imaging modalities and the histological ground truth. Autofluorescence imaging is a label-free imaging modality that captures the light emission from intrinsic fluorophores in biological tissue that are stimulated to fluoresce by external excitation. The autofluorescence images were produced by an excitation light source of 455 nm and a long-pass filter of 490 nm. The autofluorescence images were acquired from 500 to 720 nm in 10 nm increments to produce a hypercube of 23 spectral bands, and this imaging protocol was also used for the fluorescent dyes.

In addition to the two label-free modalities (HSI and autofluorescence), two dye-based fluorescence imaging modalities were acquired for SCC detection [17,18]. A fluorescently tagged glucose molecule, 2-deoxy-2-[(7-nitro-2,1,3-benzoxadiazol-4-yl) amino]-D-glucose (2-NBDG), is a dye that produces a stronger signal measured from regions with higher metabolic glucose uptake, often associated with cancer regions. The tissue specimens were incubated for 20 minutes in a 160 µM 2-NBDG solution (Cayman Chemical, Ann Arbor, MI, USA) at 37 degrees Celsius, after which tissues were rinsed in 1× phosphate buffered solution (PBS) before imaging. 

Proflavin dye images were acquired last before fixing the tissues [17,18]. Proflavin is a DNA-binding fluorescent dye that has demonstrated utility for nuclear morphology visualization in a mouse model of oral carcinogenesis [33]. The effect of proflavin staining allows greater signal-to-noise in non-keratinizing tissues, as keratin is also a target of the dye [33,34]. The tissue specimens were incubated for 120 seconds in a 0.01% proflavin solution (Sigma Aldrich, St. Louis, MO, USA) at room temperature, after which tissues were rinsed in PBS. 

## 3. Methods 

### 3.1. Histological Ground Truth and Registration

After acquiring the HSI, the fresh, ex-vivo tissue specimens were inked to preserve the optical imaging orientation, fixed in formalin, and paraffin embedded. Using a microtome, only the first, high-quality top section corresponding to the surface that was optically imaged was obtained, stained with hematoxylin and eosin, and digitized using whole-slide scanning at 40× objective [35]. The digital histology images from each specimen were annotated to outline the cancerous and normal areas by a board-certified pathologist with expertise in H and N cancer [17,18]. 

The digital histology ground truth served as the gold standard for the optical imaging modalities. The histology ground truth image was registered in a semi-automated fashion according to a previously established pipeline of deformable registration to the gross-level HSI [36]. This registration was subject to errors in tissue-deformation, uncertainty in the cancer margin with depth, and off-plane slices that in total were estimated to be about 1 mm [36]. Moreover, the variation in photon penetration depth from the optical imaging modalities and the variation in the cancer margin throughout the depth of the tissue specimens was also estimated to create another 1 to 2 mm of error in the margin, according to our previous work [37]. Therefore, a systematic and objective method for calculating classification performance was implemented by removing the area near the cancer margin in millimeter increments and reporting all values [37]. The regions near the cancer margin are both included and excluded from performance calculations because the tissue near the margin can be degenerated or have undergone pre-cancerous transformation. Here the registered cancer margin is referred to as the ‘actual TN’ margin, and mm increments estimated from the TN margin are identified. The ‘actual TN’ margin calculates performance metrics for all tissues right up to the pixels that comprise the interface of tumor and normal. For distance calculations for example, ‘TN at 1 mm’ represents that evaluation metrics are calculated from all distances up to 1 mm from the margin. The ‘TN margin at 2 mm’ is also reported, which calculates performance up to 2 mm from the margin.

### 3.2. Intra-Patient Experiments

Intra-patient experiments used a patient’s known cancer and normal specimens to train a machine learning algorithm and simulated a personalized approach for SCC detection on-the-fly in the operating room. For intra-patient experiments, linear discriminant analysis (LDA) was used in ensemble to train, validate, and test the SCC data from the same patient. Each SCC patient with all tissue types (meaning a purely normal specimen, a specimen containing only primary tumor, and a specimen of the cancer margin) was included and divided into each cohort, conventional SCC (*N* = 41) and HPV+ SCC (*N* = 6). Despite collecting 102 patients for this study, only 47 fit this distribution of all three tissue types exactly. Independently, an ensemble LDA of 500 learners was trained and validated in 5 folds from each patient’s tumor and normal samples. After each patient’s model development, the patient’s tumor-normal margin specimen was used as the testing data. The LDA method was selected because our previous work demonstrated that it outperformed other regression-based machine learning algorithms [18]. Training time for 5 cross-validated folds of one patient’s model was about one to three minutes, depending on the size of regions selected for training, which is reasonable for simulating training of a patient’s data for HSI during surgery. All statistical analyses were performed using a paired, one-tailed t-test.

### 3.3. Inter-Patient Experiments

To explore the ability of HSI and fluorescence imaging modalities to detect SCC on patients fully-independent from algorithm development, two experiments were performed. The first experiment consisted of training the CNN on primary tumor (T) and all normal (N) tissues, while testing on T and N tissues from other patients. The second experiment consisted of training on primary tumor (T) and all normal (N) tissues, while testing only tumor-involved cancer margins (TN) tissues from other patients. 

To perform these experiments, within each SCC cohort, patients were randomly divided into 5 folds, each fold served as the fully-independent testing group, while training and validation was performed on the patients in the remaining 4 folds, which allows test-level performance metrics for all patients in our dataset. For the conventional SCC cohort, each model from each fold was trained and validated on approximately 25,000 patches from 110 tissue specimens from 70 patients, and the independent testing group from each fold was approximately 50 tissues from 20 patients. This was performed once for each fold, until the entire cohort dataset, comprised of 70,000 patches from 255 tissue specimens from 88 patients was used as the testing group. For the HPV+ SCC cohort, training/validation was performed in the same fashion in 5 folds, until the entire cohort dataset of 16,000 patches from 38 tissue specimens from 14 patients was used as the testing group. All statistical analyses were performed using a paired, one-tailed t-test.

### 3.4. Convolutional Neural Network

For inter-patient experiments, a convolutional neural network (CNN) was developed to quickly and efficiently classify cancerous and normal tissues at the cancer margin. Due to the uniqueness of HSI data, the inception-v4 CNN architecture [38] was customized in several key ways to optimize the CNN to hypercube data in image-patches that were 25 × 25 × *C*, where *C* is the number of spectral bands of each HS optical modality. The full CNN architecture schematic is presented in detail in Figure S1. The CNN was developed in TensorFlow on an Ubuntu machine running NVIDIA Titan-XP GPUs [39]. The early convolutional layers were modified to handle the selected patch-size and create smaller inception blocks that would allow for faster training and classification using the CNN. Training was performed up to 50 epochs; one epoch of training data ran for up to 1 hour using HSI; and deployment of the fully-trained CNN on a single GPU to classify a new HSI scene with hundreds of patches required only 25 ± 10 seconds. The relative saliency of spectral features for correctly predicting SCC or normal in HSI, shown in Figure 1b, were extracted from the CNN using the class-activated gradients per the grad-CAM algorithm [40].

### 3.5. Image Processing and Reconstruction

For all experiments, the 10 pixels, corresponding to 0.25 mm, at the edge of each tissue were discarded for performance calculations. Since the imaging protocol for tissue specimens required using a flat imaging surface, the tissue free edges created false curvature where the tissue was too thin to provide an adequate imaging signal. Implementation of the inter-patient CNN experiments involved a patch-based approach using a sliding window of size 25 × 25 × 91 and an overlap of 13 pixels. The overlapping regions of image-patches were averaged to produce a smoother result for calculation of the performance metrics of the inter-patient experiments.

### 3.6. Evaluation

To evaluate performance of the machine learning algorithms employed in the experiments for detecting SCC, the area under the curve (AUC) of the receiver operator characteristic (ROC) curve was calculated. The AUC score was selected because it describes the accuracy at all possible thresholds of identifying the positive class and is not susceptible to errors when the classes are imbalanced. For each experiment, the optimal operating point on the ROC was calculated for the validation group data. This validation group threshold was used as the threshold for the testing group to best distinguish between cancer and normal, objectively. Using this threshold, the overall accuracy was calculated. Sensitivity, the ratio of true positives to total positive predictions, and specificity, the ratio of true negatives to total negative predictions, were also calculated and presented.

## 4. Results

### 4.1. Surgical Specimens

The accuracy of pathologist assistants in the surgical pathology department was calculated on their ability to identify the desired tissue specimen type (T, TN, or N) for research purposes. To obtain this performance, the label (prediction) given by the pathologist assistant (T, TN, or N) was compared to the ground-truth label from histology. This value is reported only to give an estimate of the difficulty of the task of positive margin and primary tumor specimen identification. It is important to note that these specimens were for research purposes only and do not attempt to reflect the accuracy of the clinical service in determining SCC during intraoperative guidance. The accuracy for identifying tissue specimens correctly was normal specimens with 92% accuracy, tumor-normal margin tissues with 95%, and primary tumor-only specimens with 60% accuracy. Figure S2a shows the breakdown by tissue specimen type. The most common reason for the misidentified tissue specimens was normal tissue in the primary tumor specimen. To calculate the accuracy, sensitivity, specificity, positive predictive value (PPV), and negative predictive value (NPV) of the specimen identification in surgical pathology, the TN tissues and predictions were separated into T and N components for calculation of true/false positives/negatives. For example, a true TN predicted as all T would count as both a true-positive and a false-positive; alternatively, a true T predicted as TN, would count as both a true-positive and a false-negative. The specificity and PPV were both 82%, the lowest metrics obtained, as the most common misidentification was tumor-margins as tumor-only, and overall an accuracy of 88% was determined for tissue type identification. Figure S2b details the complete performance metrics calculated and the number of each tissue components.

### 4.2. Intra-patient Experiments of Tumor-Involved Cancer Margins

**Figure 2:**
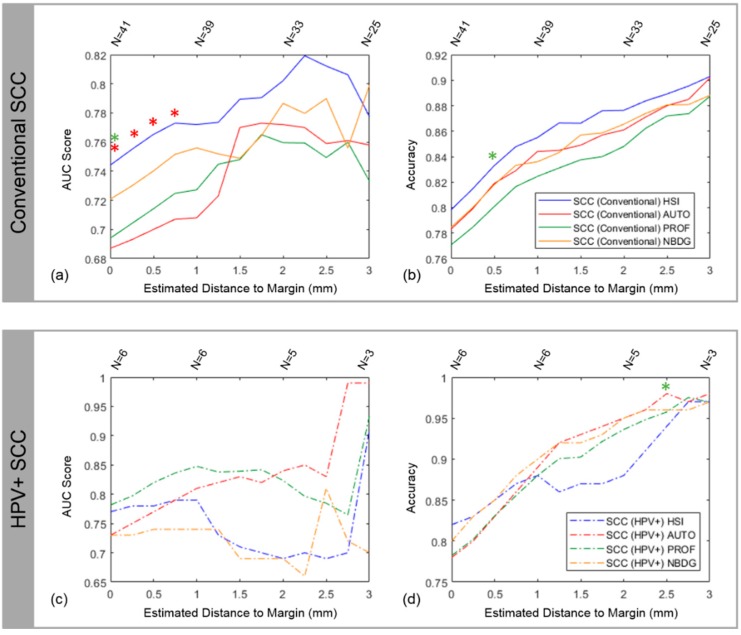
Results from intra-patient training and testing with LDA using HSI, autofluorescence, proflavin, and 2-NBDG. The results for the conventional SCC cohort are shown in (**a**) with AUC score and (**b**) with accuracy. The results for the HPV+ SCC cohort are shown in (**c**) with AUC score and (**d**) with accuracy. Statistically significant results compared to an imaging modality are indicated by a respectively colored asterisk. The sample size (N) is reported above the plots to indicate that not all distances in mm can be estimated from each tissue specimen, so the sample size decreases as the distance estimated increases, which causes noticeable jumps in the plots.

The results of reflectance-based HSI intra-patient (*N* = 47) accuracy ranged from 80 to 90% for conventional SCC and 82 to 97% for HPV+ SCC. The results of reflectance-based HSI intra-patient AUC were 0.75 to 0.82 for conventional SCC and 0.77 to 0.91 for HPV+ SCC. Table 2 shows the complete results for HSI and the fluorescence imaging modalities. The accuracy for both SCC cohorts peaked at 3 mm from the cancer margin, but the number of patients decreased with increasing distance from the cancer margin because not all distances up to 3 mm could be estimated from all tissue specimens. For the conventional SCC cohort (*N* = 41), HSI outperformed autofluorescence, proflavin, and 2-NBDG at nearly all distances from the cancer-margin and significantly outperformed proflavin and autofluorescence (*p* ≤ 0.05) close the margin (Figure 2a,b). The results for the HPV+ SCC cohort (*N* = 6) are not as conclusive as the conventional cohort because the limited number of tissue samples causes a discontinuous trend, with autofluorescence imaging being among the top performing modalities in terms of accuracy and AUC (Figure 2c,d), and the AUC obtained for autofluorescence imaging detection of HPV+ SCC was 0.99 at 3 mm, which was greater than other modalities (*p* > 0.05). In terms of accuracy, autofluorescence imaging significantly outperforms proflavin at 2.5 mm from the cancer margin (*p* < 0.05). Therefore, the label-free methods, HSI and autofluorescence, perform best for intra-patient testing at the cancer margin.

### 4.3. Inter-Patient Experiments of Tumor vs. Normal

**Figure 3:**
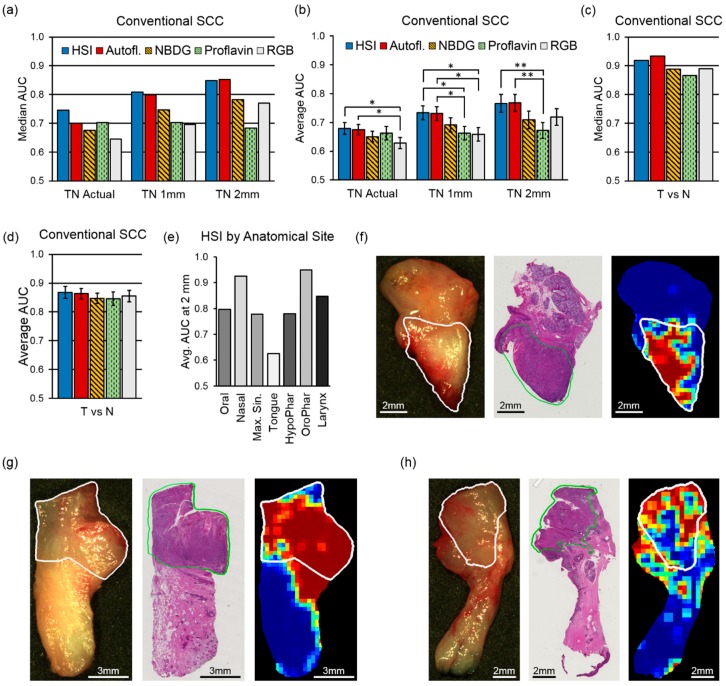
Median and average AUC results from inter-patient classification (a value of 0.5 corresponds to random guess). AUC values for the conventional SCC cohort: (**a**) Median AUC values for TN margin tissue specimens; (**b**) average AUC shown with SEM for TN margin specimens with statistical significance, shown as (*) for *p* < 0.05 and (**) for *p* < 0.01; (**c**) median AUC values for T and N whole tissue specimens; (**d**) average AUC shown with SEM for T and N whole specimens; (**e**) Average AUC at 2mm from the SCC margin using HSI across different anatomical sites; (**f–h**) representative patient examples of conventional SCC at the maxillary gingiva, nasal cavity, and larynx, respectively. From left to right: RGB made from HSI, histology ground truth, and predicted cancer heat-map. The white and green contours outline the SCC area.

**Figure 4:**
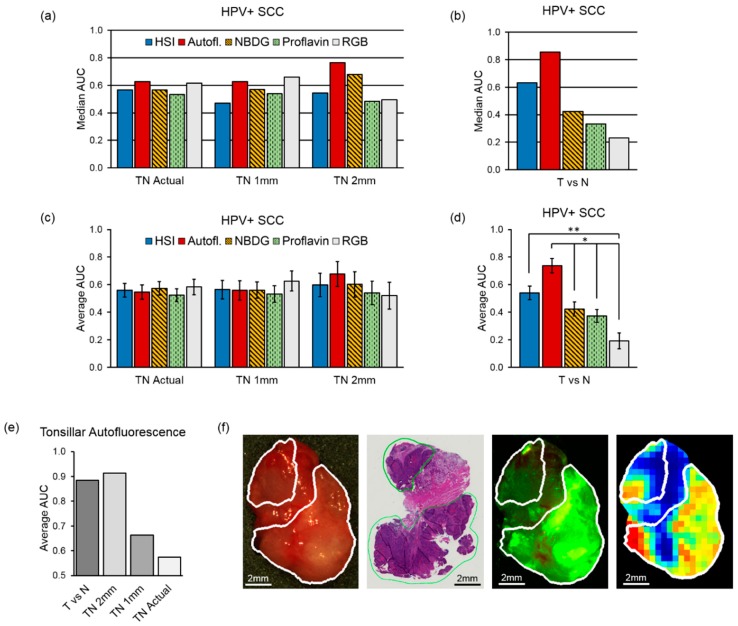
Median and average AUC results from inter-patient classification (a value of 0.5 corresponds to random guess). AUC values for the HPV+ SCC cohort: (**a**) median AUC values TN margin specimens; (**b**) median AUC values T and N whole specimens; (**c**) average AUC of TN margin specimens with SEM; (**d**) average AUC of T and N whole specimens shown with SEM and statistical significance, shown as (*) for *p* < 0.05 and (**) for *p* < 0.01; (**e**) Average AUCs of HPV+ SCC in tonsillar tissues; (**f**) Representative patient example of HPV+ SCC in tonsillar tissue from the oropharynx. From left to right: RGB made from HSI, histology ground truth, and predicted cancer heat-map. The white and green contours outline the SCC area.

For the conventional SCC cohort (*N* = 88), the results of the inter-patient experiments using only one-class specimens of the tumor-only (T) or normal-only (N) yielded similar median and average AUC scores for HSI and autofluorescence of 0.92 and 0.93 (median) and 0.87 and 0.86 (average). Both of HSI and autoluorescence outperformed the fluorescent dye-based techniques, 2-NBDG and proflavin, which had 0.88 and 0.87 (median) and both with 0.85 average AUC scores (all differences not significant with *p* > 0.05). Figure 3c,d shows the median and average AUCs for all imaging modalities for the conventional SCC cohorts. For the HPV+ SCC cohort (*N* = 14), autofluorescence imaging yielded a median AUC of 0.86 and average AUC of 0.74, which was significantly more accurate than proflavin or RGB imaging (both *p* < 0.05). For tonsillar HPV+ SCC tissues (*N* = 3), the average SCC detection was 0.89 AUC score (Figure 4b,d). The grad-CAM algorithm [40] was used to show that the salient spectral features necessary for distinguishing SCC from normal across all anatomical sites encompassed both the visible and NIR spectrum (Figure 1b). In particular, it can be observed that normal tissues have more absorption and salient spectral features in the NIR range, indicating that normal tissues have greater fat, collagen, and water content than cancer [41]. The most important spectral feature for correctly predicting SCC with HSI was the oxygenated hemoglobin peak at 560 and 565 nm, which is correlated with increased metabolic activity. Additionally, the spectral signatures and spectral feature saliency maps are shown separated by anatomical location in Figure S3.

### 4.4. Inter-patient Experiments of Tumor-Involved Cancer Margins

The results of the inter-patient experiments with HSI in the conventional SCC cohort (*N* = 88) revealed that testing on specimens of the tumor-only (T) or normal-only (N) yielded median and average AUCs greater than testing at the tumor margin (TN) at distances up to 2 mm from the margin, 0.92 and 0.87 compared to 0.85 and 0.77, respectively (Figure 3a–d). As can been seen in Figure 3b, for all distances HSI and autofluorescence outperform 2-NBDG, proflavin, and RGB imaging in average AUC score. Both HSI and autofluorescence significantly outperformed proflavin dye imaging at distances 1 mm (both *p* < 0.05) and 2 mm (both *p* < 0.01) from the cancer margin. HSI and autofluorescence significantly outperform RGB imaging in AUC at the actual cancer margin and 1 mm from the margin (all *p* < 0.05). Supplementary Table S1 shows the full results from conventional SCC with HSI at distances from the cancer margin.

The conventional SCC cohort was separated into the anatomical sites described in Table 1, and the highest average AUCs at 2mm from the cancer margin were observed in the nasal cavity (0.93), larynx (0.85), and oropharynx (0.95), while specimens from the tongue performed the lowest (Figure 3e). Representative tissue specimens from the oral cavity, nasal cavity, and larynx are classified with HSI and shown in Figure 3f–h.

For the HPV+ SCC cohort (*N* = 14) inter-patient experiments, autofluorescence imaging yielded the best results compared to the other imaging modalities at 2 mm from the cancer margin with 0.68 average AUC and 0.77 median AUC (Figure 4a,c; not significant, *p* > 0.05). For tonsillar HPV+ SCC tissues (*N* = 3), the average SCC detection with autofluorescence imaging at 2 mm from the cancer margin was 0.91 AUC (Figure 4e). A representative tissue specimen from HPV+ SCC is classified with autofluorescence imaging in Figure 4f. Supplementary Table S1 shows the full results from HPV+ SCC with autofluorescence imaging at distances from the cancer margin.

## 5. Discussion

The results of this large study of 293 tissue specimens from 102 patients with SCC show that label-free, reflectance-based hyperspectral imaging and autofluorescence imaging both outperform the fluorescent dye-based imaging methods, *i.e.,* proflavin and 2-NBDG, and this technology could aid in the detection of SCC. The fluorescent dyes employed are not specific enough to target SCC with a high signal-to-noise ratio in ex-vivo tissue specimens because of the large inter-patient variability. Proflavin allows visualization of nuclear structures, but is washed out by excessive keratin. The regional metabolic uptake of 2-NBDG to localize cancerous areas was not evident or demonstrated by the results of this ex-vivo study. Label-free HSI techniques may yield potential but the best machine learning protocols for training HSI classifier is undetermined. It may be task specific, but the results of this study show that with a large SCC HSI database, deep learning algorithms can be trained with high fidelity to work across a large number of anatomical sites in the upper aerodigestive tract. 

IPC analysis with frozen sections remains the current standard for intraoperative guidance, but it is time and labor intensive. Across all 102 patients with SCC recruited for this study, an average number of 2.1 IPCs were performed per surgery, each taking about 41 minutes in total. On average, each surgery typically investigated 3.4 tissues, each of which take about 25 minutes to report final diagnosis. The average imaging time for HSI was about 1 minute with up to 35 seconds for HSI classification using the CNN, which is significantly less than IPC. 

Detection of SCC for surgical purposes is a challenging task, whether performed by a surgeon, pathologist, or computer-aided optical imaging modality. In the literature, the accuracy of detecting positive or close margins in frozen sections ranges from 71 to 92% with sensitivity from 34 to 77% [10,11,12,13,14]. As sampling and tissue preparation is the main source of error, careful sectioning of small biopsies and vigilant communication is recommended to reduce errors during IPCs [9]. Nonetheless, significant need for guidance remains, with up to 20% to 30% of cases reported with close or positive margin results after SCC resections [10,11,12,13,14]. To this end, to put the SCC detection ability of HSI-based methods into context, we present the pathologist assistant accuracy of 88% for research purposes-only tissue identification. Since current practice is imperfect, the potential benefit of HSI for SCC detection should be evaluated on two criteria: firstly, to establish no potential harm; and secondly, to assess HSI-based intraoperative information that has clinical utility in achieving negative margins, especially considering the time advantage.

The results presented in this study using 293 specimens from 102 patients can be compared to previous pilot studies from our group. Lu et al. 2017 reported results from a small (*N* = 24) proof-of-concept study using manual ROIs that showed that training and testing on the same patient with HSI yielded an intra-patient accuracy of 89–94% and intra-patient AUC of about 0.96 [18]. Our objective and systematic approach yielded equivalent results using nearly double the intra-patient cohort (*N* = 47) for distances 1 mm beyond the cancer margin, 85–90% accuracy for conventional SCC and 88–97% accuracy for HPV+ SCC, across all anatomical tissue sites. Moreover, the AUCs obtained from 0.82 to 0.91 for conventional and HPV+ SCC cohorts at 2.25 mm from the cancer margin, importantly, are not limited by the selection of manual ROIs and include specular glare pixels. Therefore, slightly lower results are to be expected, but provide a more realistic performance estimate for HSI-based methods in the operating room. The experimental results of this study were a median AUC of 0.92 for HSI and 0.93 for autofluorescence for all conventional SCC T vs N tissues using the most patient data in an HSI study to date. The previous proof-of-concept work by Lu et al. reported an accuracy of 85% for T versus N tissues only, an accuracy of 76% for manual ROIs near the cancer-margin, and an overall average AUC of 0.88 for all tissues (T, TN, and N) for SCC at comparable tissues and anatomical sites. In comparison, in this study, for larynx, nasal cavity, and oropharyngeal SCC, we achieved AUC scores of 0.85, 0.93 and 0.95 with accuracies above 79%.

The optical imaging modalities in this study were all acquired using HSI technology, including proflavin, 2-NBDG, and autofluorescence, and all were saved as hypercubes for CNN training. Moreover, even RGB images were generated from HSI, and recent work has suggested that CNNs can recover the full HSI spectrum from RGB composites constructed from HSI [42]. Therefore, it is possible that the results from these modalities benefitted from being HS data, which is one possible explanation for not observing more statistically significant differences. The results of this study are promising at tissue sites that perform with high AUCs in both SCC cohorts. However, the results suggest that HPV+ SCC requires more data to perform well with deep learning. Therefore, the results of this study support the hypothesis that label-free HSI methods significantly outperform the dye-based methods and could provide value for clinical SCC detection.

## 6. Conclusions

In summary, reflectance-based hyperspectral imaging is label-free, non-contact, and non-ionizing, and the results of this preliminary work with 293 specimens from 102 patients demonstrates that HSI may offer utility for intraoperative SCC detection. This study is the first to report on a large SCC dataset that could be used to train a deep learning model that can predict SCC across multiple anatomical locations with high fidelity. There is a critical need to provide rapid information in the operating room for guidance during SCC resection, with errors reported up to 10–30% of missed positive and close margins in current practice. Our results show that AUCs upwards of 0.80 to 0.90 may be obtained for SCC detection with HSI-based methods in less than 2 minutes for SCC detection, and the speed can be further improved, which can save significant time as compared to intraoperative frozen section analysis.

## Figures and Tables

**Table 1:** Demographics and cancer properties for the patients recruited for this study. Values are reported for the two cohorts, conventional SCC with variants and p16+ SCC, separately and combined. Percentages for conventional are out of 88 patients, HPV+ out of 14 patients, and combined out of 102 patients. TNM staging was not available for one patient in the HPV+ cohort. All cases were M0. Tobacco history represents current or past smoking or chewing tobacco history.

Property	Conventional SCC (*N* = 88)		HPV+ SCC (*N* = 14)		All SCC (*N* = 102)	
	Number	%	Number	%	Number	%
**Demographics**						
Mean Age (y.o.)	64.5	-	58.1	-	63.6	-
Male	59	67%	12	86%	71	70%
Female	29	33%	2	14%	31	30%
Tobacco History	58	66%	5	36%	63	62%
**Primary Location**						
Oral Cavity	35	40%	0	0%	35	34%
Tongue	19	22%	0	0%	19	19%
Oropharynx	2	2%	13	93%	15	15%
Hypopharynx	4	5%	0	0%	4	4%
Larynx	19	22%	0	0%	19	19%
Nasal Cavity	4	5%	0	0%	4	4%
Maxillary Sinus	5	6%	0	0%	5	5%
Unknown	0	0%	1	7%	1	1%
**Cancer Stage**						
pT1	3	3%	2	14%	5	5%
pT2	7	8%	6	43%	13	13%
pT3	16	18%	2	14%	18	18%
pT4	62	70%	3	21%	65	64%
Avg. T Size (cm)	4.4	-	3	-	4.2	-
N+	53	60%	5	36%	58	57%
**Histologic Grade**						
G1	8	10%	-	-	8	8%
G2	60	71%	-	-	60	59%
G3	16	19%	-	-	16	16%
**IPC (Averages)**						
IPC/Surgery	2.1	-	2.0	-	2.1	-
Time/IPC (min)	41	-	42	-	41	-
Tissues/Surgery	3.5	-	2.6	-	3.4	-
Time/Tissue (min)	25	-	33	-	25	-

**Table 2:** Accuracy and area under the curve (AUC) results for the optical imaging modalities performed using the intra-patient experiments for conventional SCC and human papilloma virus (HPV+) p16-positive cohorts. Bolded values represent the greatest value in the column for each patient cohort.

Imaging Modality	SCC Cohort	1 mm		2 mm		3 mm	
		AUC	Accuracy	AUC	Accuracy	AUC	Accuracy
HSI	SCC, Conventional	**0.77**	**85%**	**0.80**	**88%**	0.78	**90%**
	SCC, HPV+	0.79	88%	0.69	88%	0.91	97%
Autofluorescence	SCC, Conventional	0.67	85%	0.72	85%	0.73	87%
	SCC, HPV+	0.81	89%	**0.84**	**95%**	**0.99**	**98%**
Proflavin	SCC, Conventional	0.73	82%	0.76	85%	0.73	89%
	SCC, HPV+	**0.85**	88%	0.82	94%	0.93	97%
2-NBDG	SCC, Conventional	0.76	84%	0.79	87%	**0.80**	89%
	SCC, HPV+	0.74	**90%**	0.69	95%	0.70	97%

## Supplementary Materials

The following are available online at https://www.mdpi.com/2072-6694/11/9/1367/s1, Figure S1: Inception V4 CNN architecture (Szegedy, 2016: Inception-v4, Inception-ResNet and the Impact of Residual Connections on Learning) with squeeze-and-excitation layers (Hu 2017: Squeeze-and-Excitation Networks) after each inception block utilizing in this work, Figure S2: Accuracy of ex-vivo tissue samples acquired, (a) The types of tissue samples acquired were all normal (N), primary tumor (T) specimen, or specimen at the tumor-normal (TN) margin. Accuracies shown are for the desired tissue type in the column; (b) The performance metrics when TN tissues and predictions were separated into T and N components for calculation of accuracy, sensitivity, specificity, PPV, and NPV, Figure S3: Spectral feature saliency from CNN gradients of correctly classified HSI (per grad-CAM) for conventional SCC and normal upper aerodigestive tract tissues separated by anatomical location, Table S1: Performance results from the best label-free HSI methods from the inter-patient experiments for each patient cohort by distance from margin (shown with ± SEM). In the conventional SCC cohort, reflectance-based HSI is presented. Additionally, the HSI results are separated by anatomical location.

## References

[1] Marur S., Forastiere A.A. (2016). Head and Neck Squamous Cell Carcinoma: Update on Epidemiology, Diagnosis, and Treatment. Mayo Clin. Proc..

[2] Bozec A., Culié D., Poissonnet G., Dassonville O. (2019). Current role of primary surgical treatment in patients with head and neck squamous cell carcinoma. Curr. Opin. Oncol..

[3] Van Keulen S., Nishio N., Fakurnejad S., Birkeland A., Martin B.A., Lu G., Zhou Q., Chirita S.U., Forouzanfar T., Colevas A.D. (2019). The Clinical Application of Fluorescence-Guided Surgery in Head and Neck Cancer. J. Nucl. Med..

[4] Joseph L.J., Goodman M., Higgins K., Pilai R., Ramalingam S.S., Magliocca K., Patel M.R., El-Deiry M., Wadsworth J.T., Owonikoko T.K. (2015). Racial disparities in squamous cell carcinoma of the oral tongue among women: A SEER data analysis. Oral Oncol..

[5] Vigneswaran N., Williams M.D. (2014). Epidemiological Trends in Head and Neck Cancer and Aids in Diagnosis. Oral Maxillofac. Surg. Clin. North Am..

[6] Gerstner A.O.H. (2010). Early detection in head and neck cancer—current state and future perspectives. Gms Curr. Top. Otorhinolaryngol. Head Neck Surg..

[7] Yao M., Epstein J.B., Modi B.J., Pytynia K.B., Mundt A.J., Feldman L.E. (2007). Current surgical treatment of squamous cell carcinoma of the head and neck. Oral Oncol..

[8] Ringash J. (2015). Survivorship and Quality of Life in Head and Neck Cancer. J. Clin. Oncol..

[9] Baddour H.M., Magliocca K.R., Chen A.Y. (2016). The importance of margins in head and neck cancer. J. Surg. Oncol..

[10] Dinardo L.J., Lin J., Karageorge L.S., Powers C.N. (2000). Accuracy, Utility, and Cost of Frozen Section Margins in Head and Neck Cancer Surgery. Laryngoscope.

[11] Ribeiro N., Godden D., Wilson G., Butterworth D., Woodwards R. (2003). Do frozen sections help achieve adequate surgical margins in the resection of oral carcinoma?. Int. J. Oral Maxillofac. Surg..

[12] Du E., Ow T.J., Lo Y., Gersten A., Schiff B.A., Tassler A.B., Smith R.V. (2016). Refining the utility and role of Frozen section in head and neck squamous cell carcinoma resection. Laryngoscope.

[13] Layfield E.M., Schmidt R.L., Esebua M., Layfield L.J. (2018). Frozen Section Evaluation of Margin Status in Primary Squamous Cell Carcinomas of the Head and Neck: A Correlation Study of Frozen Section and Final Diagnoses. Head Neck Pathol..

[14] Black C., Marotti J., Zarovnaya E., Paydarfar J. (2006). Critical evaluation of frozen section margins in head and neck cancer resections. Cancer.

[15] Gao R.W., Teraphongphom N.T., Berg N.S.V.D., Martin B.A., Oberhelman N.J., Divi V., Kaplan M.J., Hong S.S., Lu G., Ertsey R. (2018). Determination of Tumor Margins with Surgical Specimen Mapping Using Near-Infrared Fluorescence. Cancer Res..

[16] Crowson M.G., Ranisau J., Eskander A., Babier A., Xu B., Kahmke R.R., Chen J.M., Chan T.C.Y. (2019). A contemporary review of machine learning in otolaryngology-head and neck surgery. Laryngoscope.

[17] Fei B., Lu G., Wang X., Zhang H., Little J.V., Patel M.R., Griffith C.C., El-Diery M.W., Chen A.Y. (2017). Label-free reflectance hyperspectral imaging for tumor margin assessment: A pilot study on surgical specimens of cancer patients. J. Biomed. Opt..

[18] Lu G., Little J.V., Wang X., Zhang H., Patel M.R., Griffith C.C., El-Deiry M.W., Chen A.Y., Fei B. (2017). Detection of Head and Neck Cancer in Surgical Specimens Using Quantitative Hyperspectral Imaging. Clin. Cancer Res..

[19] Farah C.S., Fox S.A., Dalley A.J. (2018). Integrated miRNA-mRNA spatial signature for oral squamous cell carcinoma: A prospective profiling study of Narrow Band Imaging guided resection. Sci. Rep..

[20] Farah C. (2018). Narrow Band Imaging-guided resection of oral cavity cancer decreases local recurrence and increases survival. Oral Dis..

[21] Lu G., Fei B. (2014). Medical hyperspectral imaging: A review. J. Biomed. Opt..

[22] Halicek M., Fabelo H., Ortega S., Callico G.M., Fei B. (2019). In-Vivo and Ex-Vivo Tissue Analysis through Hyperspectral Imaging Techniques: Revealing the Invisible Features of Cancer. Cancers.

[23] Shapey J., Xie Y., Nabavi E., Bradford R., Saeed S.R., Ourselin S., Vercauteren T. (2019). Intraoperative multispectral and hyperspectral label-free imaging: A systematic review of in vivo clinical studies. J. Biophotonics.

[24] Fabelo H., Halicek M., Ortega S., Shahedi M., Szolna A., Piñeiro J.F., Sosa C., O’Shanahan A.J., Bisshopp S., Espino C. (2019). Deep Learning-Based Framework for In Vivo Identification of Glioblastoma Tumor using Hyperspectral Images of Human Brain. Sensors.

[25] Fabelo H., Ortega S., Szolna A., Bulters D., Pineiro J.F., Kabwama S., J-O’Shanahan A., Bulstrode H., Bisshopp S., Kiran B.R. (2019). In-Vivo Hyperspectral Human Brain Image Database for Brain Cancer Detection. IEEE Access.

[26] Baltussen E.J.M., Kok E.N.D., De Koning S.G.B., Sanders J., Aalbers A.G.J., Kok N.F.M., Beets G.L., Flohil C.C., Bruin S.C., Kuhlmann K.F.D. (2019). Hyperspectral imaging for tissue classification, a way toward smart laparoscopic colorectal surgery. J. Biomed. Opt..

[27] Leavesley S.J., Walters M., Lopez C., Baker T., Favreau P.F., Rich T.C., Rider P.F., Boudreaux C.W. (2016). Hyperspectral imaging fluorescence excitation scanning for colon cancer detection. J. Biomed. Opt..

[28] Halicek M., Little J.V., Wang X., Patel M.R., Griffith C.C., Chen A.Y., Fei B. (2018). Tumor Margin Classification of Head and Neck Cancer Using Hyperspectral Imaging and Convolutional Neural Networks. Proc. SPIE Int. Soc. Opt. Eng..

[29] Halicek M., Lu G., Little J.V., Wang X., Patel M., Griffith C.C., El-Deiry M.W., Chen A.Y., Fei B. (2017). Deep convolutional neural networks for classifying head and neck cancer using hyperspectral imaging. J. Biomed. Opt..

[30] Halicek M., Little J.V., Wang X., Chen A.Y., Fei B. (2019). Optical biopsy of head and neck cancer using hyperspectral imaging and convolutional neural networks. J. Biomed. Opt..

[31] Manni F., Van Der Sommen F., Zinger S., Kho E., De Koning S.G.B., Ruers T.J.M., Shan C., Schleipen J., De With P.H.N. (2019). Automated tumor assessment of squamous cell carcinoma on tongue cancer patients with hyperspectral imaging. Proc. SPIE.

[32] Trajanovski S., Shan C., Weijtmans P.J.C., de Koning S.G.B., Ruers T.J.M. Tumor Semantic Segmentation in Hyperspectral Images using Deep Learning. Proceedings of the 2nd International Conference on Medical Imaging with Deep Learning.

[33] Hellebust A., Rosbach K., Wu J.K., Nguyen J., Gillenwater A., Vigneswaran N., Richards-Kortum R. (2013). Vital-dye-enhanced multimodal imaging of neoplastic progression in a mouse model of oral carcinogenesis. J. Biomed. Opt..

[34] Thekkek N., Muldoon T., Polydorides A.D., Maru D.M., Harpaz N., Harris M.T., Anandasabapathy S. (2012). Vital-dye enhanced fluorescence imaging of GI mucosa: Metaplasia, neoplasia, inflammation. Gastrointest. Endosc..

[35] Halicek M., Shahedi M., Little J.V., Chen A.Y., Myers L.L., Sumer B.D., Fei B. (2019). Detection of squamous cell carcinoma in digitized histological images from the head and neck using convolutional neural networks. Proc. SPIE.

[36] Halicek M., Little J.V., Wang X., Chen Z.G., Patel M.R., Griffith C.C., El-Deiry M.W., Saba N.F., Chen A.Y., Fei B. (2018). Deformable Registration of Histological Cancer Margins to Gross Hyperspectral Images using Demons. Proc. SPIE Int. Soc. Opt. Eng..

[37] Halicek M., Fabelo H., Ortega S., Little J.V., Wang X., Chen A.Y., Callico G.M., Myers L., Sumer B.D., Fei B. (2019). Hyperspectral imaging for head and neck cancer detection: Specular glare and variance of the tumor margin in surgical specimens. J. Med Imaging.

[38] Szegedy C., Ioffe S., Vanhoucke V., Alemi A. Inception-v4, Inception-ResNet and the Impact of Residual Connections on Learning. Proceedings of the Thirty-First AAAI Conference on Artificial Intelligence.

[39] Abadi M., Agarwal A., Barham P., Brevdo E., Chen Z., Citro C., Corrado G.S., Davis A., Dean J., Devin M. (2015). TensorFlow: Large-scale Machine Learning on Heterogeneous Systems. https://www.tensorflow.org.

[40] Selvaraju R.R., Cogswell M., Das A., Vedantam R., Parikh D., Batra D. Grad-CAM: Visual Explanations from Deep Networks via Gradient-Based Localization. Proceedings of the IEEE International Conference on Computer Vision (ICCV).

[41] Sekar S.K.V., Bargigia I., Mora A.D., Taroni P., Ruggeri A., Tosi A., Pifferi A., Farina A. (2017). Diffuse optical characterization of collagen absorption from 500 to 1700 nm. J. Biomed. Opt..

[42] Shi Z., Chen C., Xiong Z., Liu D., Wu F. HSCNN+: Advanced CNN-Based Hyperspectral Recovery from RGB Images. Proceedings of the IEEE/CVF Conference on Computer Vision and Pattern Recognition Workshops (CVPRW).

