# Supplementary material for: Hyperspectral Imaging of Head and Neck Squamous Cell Carcinoma for Cancer Margin Detection in Surgical Specimens from 102 Patients Using Deep Learning
Source: Cancers (Basel). 2019 Sep 14;11(9):1367. doi: 10.3390/cancers11091367 (PMC6769839; doi:10.3390/cancers11091367)
Supplement: Supplementary file 1 [file cancers-11-01367-s001.pdf]

SupplementaryMaterials

Hyperspectral Imaging of Head and Neck Squamous Cell Carcinoma for Cancer Margin Detection in Surgical Specimens from 102 Patients Using Deep Learning

Martin Halicek, James D. Dormer, James V. Little, Amy Y. Chen, Larry Myers, Baran D. Sumer and Baowei Fei

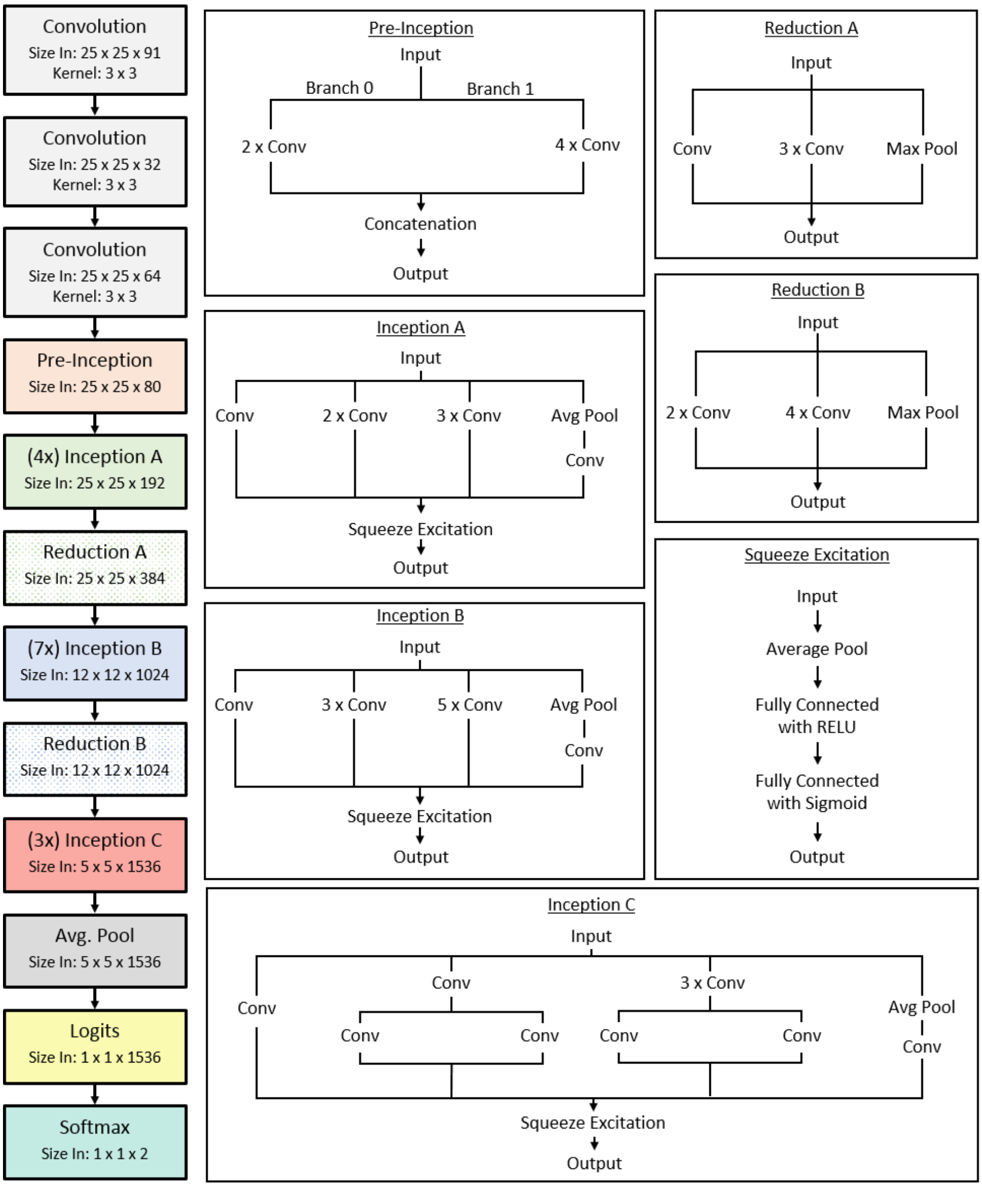

Figure S1. Inception V4 CNN architecture (Szegedy, 2016: Inception-v4, Inception-ResNet and the Impact of Residual Connections on Learning) with squeeze-and-excitation layers (Hu 2017: Squeeze-and-Excitation Networks) after each inception block utilizing in this work. “Same” padding was used in all layers except for the Reduction Blocks, which used “valid” padding. The early convolution layers were modified from the traditional inception V4 architecture to account for the hyperspectral nature of the data used.

(a)

Histology Ground Truth \ Surgical Path Prediction	T	TN	N	Total
**T**	**59**	2	0	61
**TN**	37	**94**	8	139
**N**	2	3	**88**	93
Total	98	99	96	293
Accuracy	60%	95%	92%	82%

(b)

Histology Ground Truth \ Surgical Path Prediction	T	N	Total	
T	192	10	202	Sens.: 95%
N	42	193	235	Spec.: 82%
Total	234	203	437	
	PPV: 82%	NPV: 95%		Acc.: 88%

Figure S2. Accuracy of ex-vivo tissue samples acquired. (**a**) The types of tissue samples acquired were all normal (N), primary tumor (T) specimen, or specimen at the tumor-normal (TN) margin. Accuracies shown are for the desired tissue type in the column. (**b**) The performance metrics when TN tissues and predictions were separated into T and N components for calculation of accuracy, sensitivity, specificity, PPV, and NPV. For example, a true TN predicted as all T would count as both a true-positive and a false-positive; alternatively, a true T predicted as TN, would count as both a true-positive and a false-negative. Therefore, the 139 true TNs and 5 false predicted TNs are double counted, so the specimen total is now 437 instead of 293 specimens.

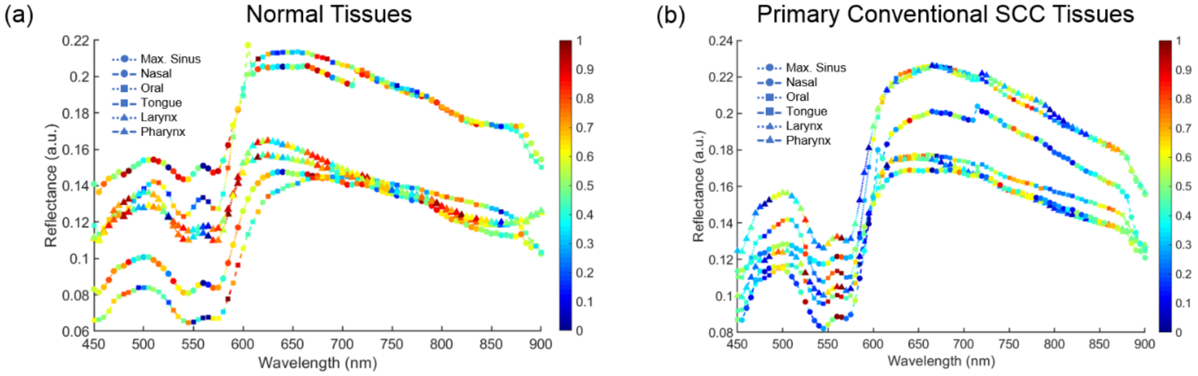

Figure S3. Spectral feature saliency from CNN gradients of correctly classified HSI (per grad-CAM) for conventional SCC and normal upper aerodigestive tract tissues separated by anatomical location. (**a**): Spectral signatures with spectral feature importance for normal tissues from the maxillary sinus, nasal cavity, oral cavity, tongue, larynx, and pharynx. Color of symbol represents the relative importance of the spectral feature for assigning the correct label of the class (0 – blue, low saliency; 1 – red, high saliency). (**b**): Spectral signatures with spectral feature importance for primary conventional SCC tissues from the maxillary sinus, nasal cavity, oral cavity, tongue, larynx, and pharynx.

Table S1. Performance results from the best label-free HSI methods from the inter-patient experiments for each patient cohort by distance from margin (shown with ± SEM). In the conventional SCC cohort, reflectance-based HSI is presented. Additionally, the HSI results are separated by anatomical location. For the HPV+ SCC cohort, autofluorescence is presented, which all came from the pharynx.

Cohort / Method	Median AUC	Average AUC	Accuracy	Sensitivity	Specificity
**Conventional SCC Reflectance-based HSI**					
TN Actual	0.75	0.68 ± 0.02	60 ± 2%	60 ± 4%	54 ± 3%
TN 1mm	0.81	0.73 ± 0.02	66 ± 2%	60 ± 4%	61 ± 4%
TN 2 mm	0.85	0.77 ± 0.03	64 ± 3%	65 ± 4%	59 ± 5%
**Conventional SCC Reflectance-based HSI**					
Oral Cavity, TN 2mm	0.81	0.79 ± 0.04	63 ± 5%	71 ± 8%	49 ± 8%
Tongue, TN 2mm	0.78	0.64 ± 0.07	61 ± 7%	57 ± 9%	53 ± 9%
Nasal Cavity, TN 2mm	0.98	0.93 ± 0.06	79 ± 11%	69 ± 17%	73 ± 24%
Max. Sinus, TN 2mm	0.95	0.78 ± 0.18	58 ± 19%	93 ± 5%	52 ± 18%
Larynx, TN 2mm	0.86	0.85 ± 0.05	79 ± 5%	69 ± 11%	71 ± 9%
Hypopharynx, TN 2mm	0.84	0.78 ± 0.13	42 ± 9%	20 ± 14%	99 ± 1%
Oropharynx, TN 2mm	0.95	0.95 ± 0.001	95 ± 4%	49 ± 49%	78 ± 22%
**HPV+ SCC Autofluorescence**					
TN Actual	0.63	0.55 ± 0.05	60 ± 4%	45 ± 6%	65 ± 6%
TN 1mm	0.63	0.56 ± 0.07	65 ± 5%	49 ± 8%	62 ± 8%
TN 2 mm	0.77	0.68 ± 0.09	63 ± 7%	64 ± 8%	60 ± 9%

© 2019 by the authors. Licensee MDPI, Basel, Switzerland. This article is an open access article distributed under the terms and conditions of the Creative Commons Attribution (CC BY) license (http://creativecommons.org/licenses/by/4.0/).
